# The effects of tourism e-commerce live streaming features on consumer purchase intention: The mediating roles of flow experience and trust

**DOI:** 10.3389/fpsyg.2022.995129

**Published:** 2022-08-26

**Authors:** Xiaoli Liu, Lei Zhang, Qian Chen

**Affiliations:** ^1^Library, Zhejiang Gongshang University, Hangzhou, China; ^2^Publicity Department, China Jiliang University, Hangzhou, China; ^3^School of Tourism and Urban-Rural Planning, Zhejiang Gongshang University, Hangzhou, China

**Keywords:** tourism e-commerce, live streaming, interactivity, authenticity, entertainment, flow experience, trust, purchase intention

## Abstract

Given that tourism e-commerce live streaming has become an important driver of tourism development after the outbreak of Covid-19 but limited attention has been paid to this area, this study examines the impacts of tourism e-commerce live streaming features (interactivity, authenticity, and entertainment) on the consumers’ purchase intention from the perspectives of consumers’ flow experience and trust based on the SOR theory. The authors collected survey data from 357 tourism e-commerce live streaming consumers and used the structural equation model to test the research model. The results reveal that interactivity and authenticity positively affect tourism e-commerce live streaming consumers’ purchase intention, but entertainment does not influence purchase intention positively; interactivity, authenticity, and entertainment each positively affects tourism e-commerce live streaming consumers’ flow experience and trust; both flow experience and trust positively affect tourism e-commerce live streaming consumers’ purchase intention; both flow experience and trust have mediating effects on the relationships between tourism e-commerce live streaming features and consumers’ purchase intention. This study extends existing theoretical research on tourism e-commerce live streaming and provides some managerial implications for tourism enterprises and streamers.

## Introduction

With the rapid development of digital technology, e-commerce live streaming has become a new business model (Wongkitrungrueng and Assarut, 2020; Sun et al., 2022). After the outbreak of COVID-19 pandemic in early 2020, the large-scale travel restrictions further pushed up the popularity of e-commerce live streaming. In China, the scale of e-commerce live streaming users was 464 million in 2021, with an increase of 75.79 million than in December 2020 (CNNIC, 2022). In the field of tourism, the information needs and consumption habits of tourists are changing due to the impact of COVID-19. Different sectors of tourism industry applied high tech or live streaming approach to respond to COVID-19. For example, facial recognition and smart cameras were used in gambling industry (Liu et al., 2021b), digital technology was applied in hospitality industry in Macao (Liu et al., 2021c), and live streaming was used in tourism industry (Liu et al., 2022). Tourism e-commerce live streaming is developing rapidly (Deng et al., 2021; Xie et al., 2022). In China, online travel agents (OTAs), such as Ctrip, Mafengwo, and Tuniu, are accelerating the development of live streaming and exploring the business model innovation of “tourism + live streaming” through e-commerce live streaming and virtual tourism, such as the “Boss Live Session” activity initiated by Ctrip.

At the same time, e-commerce live streaming research has become a research hotspot (Ang et al., 2018; Zhang et al., 2020, 2022). E-commerce live streaming attracts consumers through instant interaction and vivid product display (Tong, 2017; Ang et al., 2018; Liu et al., 2020c). E-commerce live streaming can deliver richer information to consumers than posts that mainly convey product information through text and pictures(Yu and Zheng, 2022).Serving the more detailed and vertical needs of consumers, e-commerce live streaming attracts potential consumers, improves the conversion rate, and generates faster sales (Hu and Chaudhry, 2020). It can both improve the conversion rate of both physical and virtual stores, and expose the brand to the public (Xue and Liu, 2022). Previous studies have shown that e-commerce live streaming consumer’ purchase intentions can be influenced by live streaming strategy (Zhang et al., 2020), IT affordances (Sun et al., 2019), interaction (Li et al., 2021; Zhang et al., 2021b), and social presence (Ang et al., 2018; Chen and Liao, 2022). However, little research has been devoted to studying the impacts of e-commerce live streaming features on consumer’s purchase intention systematically, and there are still very few studies on tourism e-commerce live streaming (Deng et al., 2021; Qiu et al., 2021; Lin et al., 2022).

Tourism e-commerce live streaming promotes the marketing of tourism industry, taps the online consumption potential of tourists, and achieves the synergistic development of tourism online and offline (Zhang et al., 2021b; Xie et al., 2022). Despite the growing popularity of tourism live streaming, little research has been devoted to studying the impacts of tourism e-commerce live streaming features on consumer’s purchase intention (Lv et al., 2022). Based on a review of studies pertaining to e-commerce live streaming, we proposed three core features of this form of communication: interactivity (Xue et al., 2020; Kang et al., 2021), authenticity (Tong, 2017), and entertainment (Chen and Lin, 2018). The tourism e-commerce streamer interacts with the consumers, and the consumers can also interact with each other through pop-ups or other forms, forming an open virtual community centered on the streamer. Compared with the traditional tourism e-commerce marketing model in which consumers initiate the consultations, the interaction in the e-commerce live streaming is intuitive, instantaneous, and interactive, changing from traditional passive service to active guidance and creating a more realistic tourism shopping scenario. At the same time, tourism streamers show tourism products and exchange the information of the products through live streaming, helping tourism consumers establish an authentic perception of the tourism products. That is, tourism e-commerce live streaming creates a face-to-face shopping scenario in comparison with traditional tourism e-commerce. Thus, the perceived authenticity of tourism products is stronger, which helps to enhance the consumer’s trust (Jiménez-Barreto et al., 2020). Another feature of tourism e-commerce live streaming is entertainment. Compared with e-commerce, the entertainment in e-commerce live streaming comes not only from the perception of the shopping experience, but also from the live streaming content and participation process, which is more conducive to the consumer’s flow experience. However, little research has been devoted to regarding interactivity, authenticity, and entertainment as the features of e-commerce live streaming to study their impacts on consumer’s flow experience and trust in an empirical study, especially in the field of tourism.

To fill these gaps, based on the stimulus-organism-response (SOR) model, we used tourism e-commerce live streaming features (interactivity, authenticity, and entertainment) as stimulus variables (S), flow experience and trust as organism variables (O), and tourism consumers’ purchase intention as the response variable (R), to explore the influence mechanism of tourism e-commerce live streaming features on tourism consumers’ purchase intention. We aim to enrich and deepen the research on the formation mechanism of tourism consumers’ purchase intention in the context of tourism e-commerce live streaming theoretically, and practically provide guidance to enhance tourism consumers’ purchase intention and help to realize the integrated development of tourism industry online and offline.

## Theoretical background and hypothesis development

### SOR theory

**Figure 1:**
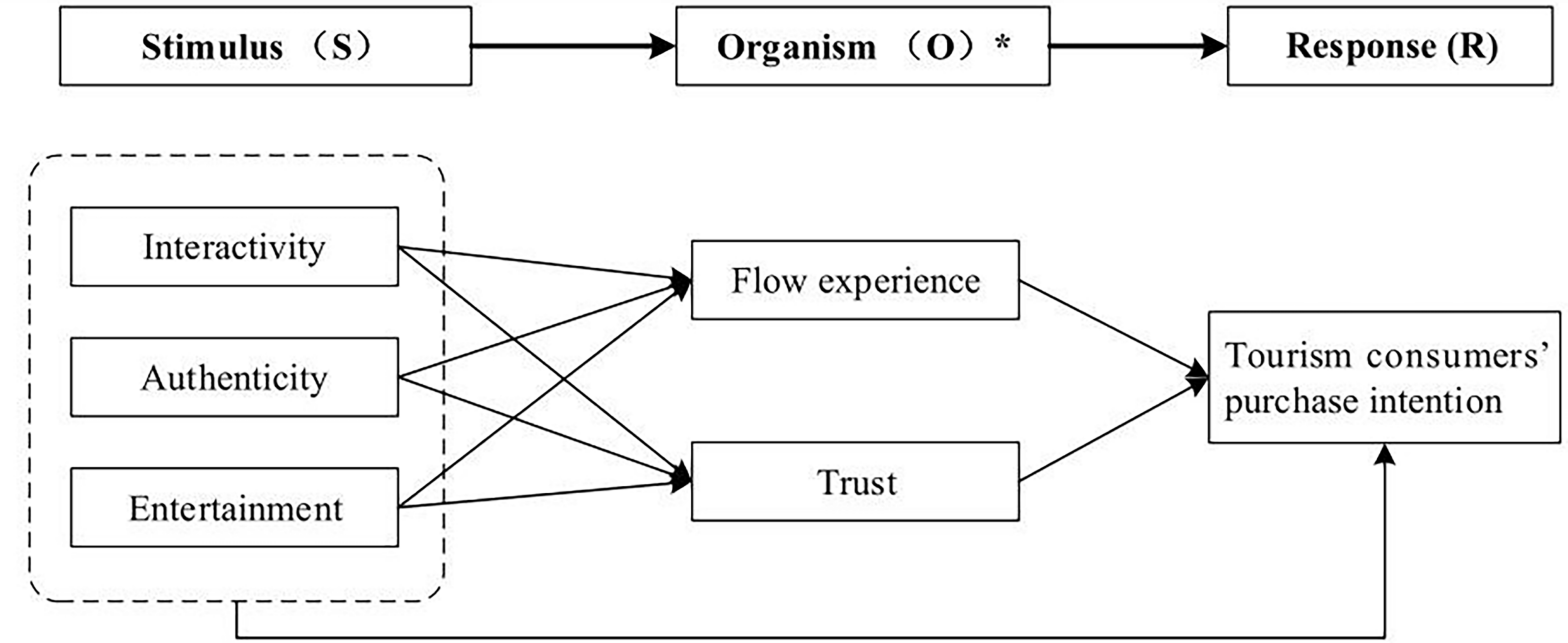
Research model. ^*^, additional analysis is conducted to examine the mediating effect of organism.

To study the influence of the external environment on individual behavior, the stimulus-organism-response (SOR) theoretical model was proposed in the field of environmental psychology (Mehrabian and Russell, 1974). In this context, stimulus (S) refers to external environmental factors that can act on an individual’s cognition and emotion (O) and ultimately elicit a behavioral response (R). A few studies has applied the SOR model in the research of e-commerce live streaming consumers. For example, Xu et al. (2020) employed the SOR framework to investigate contextual and environmental stimuli effects (streamer attractiveness, para-social interactions, and information quality) from a e-commerce live streaming context on viewer’s cognitive and emotional states (cognitive assimilation and arousal) and their subsequent responses (hedonic consumption, impulsive consumption, and social sharing); Guo J. et al. (2021) applied the SOR framework to examine the impact of live streaming feature on the consumers’ cross-border purchase intention from the perspectives of consumers’ overall perceived value and overall perceived uncertainty. However, Xu et al. (2020) did not pay attention to the roles of the e-commerce live streaming features, while Guo J. et al. (2021) regarded the live streaming feature as a concept and did not subdivide the live streaming feature. In addition, since tourism products have special features (e.g., high unit price, low purchase frequency, intangibility, and non-transferability) that are different from general products (Xie et al., 2022), it is necessary to study on tourism e-commerce live streaming and the consumers’ psychology. In this study, tourism e-commerce live streaming features (interactivity, authenticity, and entertainment) were selected to assess the contextual and environmental stimuli, flow experience and trust were selected to assess the internal states of tourism consumers, and tourism customers’ purchase intention were selected to assess their responses. The research model is shown in Figure 1.

### The effects of tourism e-commerce live streaming features

Interactivity means that consumers can communicate and exchange information with the information source, emphasizing the two-way communication. When watching tourism e-commerce live streaming, viewers can consult and give gifts to the streamer, express their opinions, and communicate with other viewers through pop-ups. The streamers also actively communicate with their viewers in addition to presenting the products (Liu et al., 2020c; Wang and Liu, 2022). In the process of live streaming, the frequent interactions between the streamer and consumers make consumers feel temporarily detached from reality, forget about worries, and have a sense of immersion (Liu et al., 2020a). The interactive communication between streamers and consumers generates an interactive feedback signal to customers, which can produce a powerful psychological implication to customers and increase their trust in the streamers (Chen et al., 2021). A high level of interaction between streamers and consumers can lead to cognitive and emotional changes of consumers, enhance consumers’ understanding of the streamers and products, and thus increase trust, which ultimately influences consumers’ purchase intentions (Hou et al., 2020; Zhang et al., 2021a). Liu et al. (2021a) emphasized the importance of interactivity in tourism live streaming. As such, we believe that tourism e-commerce live streaming with strong interactivity can stimulate consumers’ flow experience, trust, and purchase intention. Based on this, this paper proposes the following hypotheses:

*H1a*: Interactivity of tourism e-commerce live streaming positively affects consumers’ flow experience.

*H1b*: Interactivity of tourism e-commerce live streaming positively affects consumers’ trust.

*H1c*: Interactivity of tourism e-commerce live streaming positively affects consumers’ purchase intention.

Authenticity refers to the individual’s evaluation of the truthfulness of the information received. In the traditional tourism marketing, there is a risk of excessive embellishment, lens switching, or image manipulation of pictures (Zhang et al., 2021a). Customers cannot see the real products (Lu et al., 2016), making them vulnerable to be cheated by inauthentic and beautified information and may hinder trust-building (Escobar-Rodríguez and Bonsón-Fernández, 2017; Guo L. et al., 2021). In tourism e-commerce live streaming, the live streaming process is live and instant, without camera switching. It is a complete presentation of the whole tourism scene and products, with a strong sense of live immersion. In the process of live streaming, the streamers give real descriptions and effective evaluations of the products and offer purchase suggestions, which increase customers’ interest in watching the live streaming (Li et al., 2021). Therefore, tourism e-commerce live streaming with authenticity will attract consumers and bring them into a specific scenario, thus creating a positive emotional experience for consumers. Tong (2017) emphasized that the authenticity of a webcast enhanced customer engagement and trust. Zhang et al. (2021a) showed that live streaming authenticity not only had a positive impact on consumer perceptions, but also influenced consumers’ purchase intentions. Liu et al. (2021a) argued that authenticity was crucial in tourism live streaming. As such, we believe that tourism e-commerce live streaming with strong authenticity can stimulate consumers’ flow experience, trust, and purchase intention. Based on this, this paper proposes the following hypotheses:

*H2a*: Authenticity of tourism e-commerce live streaming positively affects consumers’ flow experience.

*H2b*: Authenticity of tourism e-commerce live streaming positively affects consumers’ trust.

*H2c*: Authenticity of tourism e-commerce live streaming positively affects consumers’ purchase intention.

Entertainment refers to the degree of pleasure felt by consumers during the process of watching live streaming, with the aim of satisfying consumers’ pleasure psychology. Viewers tend to use media to relieve stress for entertainment (Chen and Lin, 2018). To a large extent, consumers participate in consumption for the purpose of personal relaxation and stress relief (Wang et al., 2020). Entertainment is reflected in the lively and interesting topics started by the streamer, and a series of entertaining activities held by the shopping platform or the streamer, such as regular lottery, virtual red envelope distribution, and thumb-up, etc. (Liu et al., 2020c). Meanwhile, the creative pop-up messages posted by the viewers and the hover animation of the live streaming window also increase the entertainment of the e-commerce live streaming (Yu and Xu, 2017). Entertainment in live streaming can significantly influence consumers’ flow experience, perceived value, and usage attitude (Chen and Lin, 2018; Cao et al., 2022), and also increase the emotional connection between the streamer and consumers (Hilvert-Bruce et al., 2018). Previous studies showed that entertainment had a significant effect on tourist trust (Pujiastuti et al., 2017), social media brand trust (Zhang et al., 2022), and purchase intention (Ma et al., 2022). From this, it can be hypothesized that tourism e-commerce live streaming with strong entertainment can stimulate consumers’ flow experience, trust, and purchase intention. Based on this, this paper proposes the following hypotheses:

*H3a*: Entertainment of tourism e-commerce live streaming positively affects consumers’ flow experience.

*H3b*: Entertainment of tourism e-commerce live streaming positively affects consumers’ trust.

*H3c*: Entertainment of tourism e-commerce live streaming positively affects consumers’ purchase intention.

### The effect of flow experience

In the online context, flow leads users to become completely engaged in online tasks and interested to continue these activities. As the consumer experience quality is higher, the perceived value is higher and consumers are more willing to participate (Prentice et al., 2019; Chen et al., 2022b). E-commerce live streaming enables consumers to enjoy a sense of freedom, control and participation, and a better consumption experience, which can lead to consumers’ willingness to purchase (Feng and Lu, 2020). Flow experience represents an intense involvement that leads to high psychological engagement such as satisfaction and loyalty for virtual world users (Barker, 2016). Gao and Bai (2014) noted that flow experience affected consumers’ behavioral intention, such as the likelihood to purchase from the website. The online students’ flow experience has a significant relationship with continuous intention (Zhao and Khan, 2022). In social commerce, consumers who have experienced flow are likely to participate in social commerce activities (Zhang et al., 2014), which affects consumers’ purchase intention (Xu et al., 2022). From this, it can be presumed that tourism e-commerce live streaming consumers with a stronger flow experience are more likely to generate purchase intentions. Based on this, this paper proposes the following hypothesis:

*H4:* Flow experience positively affects tourism e-commerce live streaming consumers’ purchase intention.

### The effect of trust

Perceived trust refers to the degree of consumers’ trust in the tourism e-commerce live streaming streamer and the products recommended by the streamer. Consumers tend to make purchase decisions in a short period of time and with limited rationality because of perceived trust (Liu and Shi, 2020; Liu et al., 2021e). In e-commerce live streaming, trust helps to reduce various transaction costs (Feng and Lu, 2020), and reduce consumers’ perceived risk and uncertainty about the streamers and products, makeing consumers actively participate in online transactions (Liu et al., 2020c; Guo J. et al., 2021). Prior studies demonstrated that trust had an important effect on consumer behavior (Nadeem et al., 2020; Guo J. et al., 2021). Alkhalifah (2022) confirmed that trust in social commerce influenced behavior intention. Dong et al. (2022) demonstrated that live-streaming e-commerce with high-quality would increase consumers’ green trust and, thus, strengthen green purchase intention. In the context of tourism, tourist trust is widely accepted to play an important role in influencing their behavior intentions (Iranmanesh et al., 2018; Han et al., 2021). It is difficult for consumers to make purchase decisions in tourism e-commerce live streaming because of high uncertainty and perceived risk, but perceived trust can help consumers reduce their decision costs and thus generate purchase intentions (Lu and Chen, 2021). From this, it can be hypothesized that consumers with stronger trust in tourism e-commerce live streaming are more likely to generate purchase intention. Based on this, this paper proposes the following hypothesis:

*H5*: Trust positively affects tourism e-commerce live streaming consumers’ purchase intention.

### The mediating role of flow experience

Frequent interactions in e-commerce live streaming make consumers temporarily detach from reality and immerse themselves in the live streaming environment, forgetting their worries and generating a flow experience (Liu et al., 2020a). The streamer displays the product realistically and evaluate it effectively, give purchase suggestions, and increase customers’ interest in the product when watching the live broadcast (Li et al., 2021). Entertainment in e-commerce live streaming can significantly influence consumers’ flow experience, perceived value, and attitude (Chen and Lin, 2018). Consumers’ flow experience has an impact on attitudes, and when consumers are immersed in the live streaming environment, they want to participate unconsciously and are stimulated by the streamer to purchase (Huang et al., 2021). Arghashi and Yuksel (2022) demonstrated that consumers’ flow experience mediated the relationship of interactivity and trust in AR apps. From this, it can be hypothesized that consumers obtain flow experience by watching tourism e-commerce live streaming and generate purchase intention under the influence of flow experience. Based on this, this paper proposes the following hypotheses:

*H6a*: Flow experience has a mediating effect between interactivity and purchase intention in tourism e-commerce live streaming.

*H6b*: Flow experience has a mediating effect between authenticity and purchase intention in tourism e-commerce live streaming.

*H6c*: Flow experience has a mediating effect between entertainment and purchase intention in tourism e-commerce live streaming.

### The mediating role of trust

In e-commerce live streaming, interactivity can form an intimate relationship between the streamer and consumers and increase consumers’ perceived trust (Wei et al., 2022). Authenticity can enhance viewers’ understanding of the products, reduce perceived risk, and promote trust (Tong, 2017). Entertainment can increase consumers’ curiosity about the streamer and the product, and enhance their desire to participate in the live streaming, which leads to positive evaluation of the product and the streamer (Wongkitrungrueng and Assarut, 2020). Perceived trust is an important factor to maintain loyalty and is the foundation of online shopping. Trust comes from the daily interaction between streamers and viewers, the professional competence of streamers, etc. (Zhang et al., 2021a). According to Alalwan et al. (2019) and Kim and Park (2013), trust mediates the relationships between s-commerce dimensions and consumers’ value co-creation, and between the characteristics of s-commerce and purchase intention. Liu et al. (2021d) confirmed that social support had a direct positive effect on s-commerce purchase intention, and that social trust partially mediated the relationship. From this, it can be hypothesized that consumers generate trust by watching tourism e-commerce live streaming and generate purchase intention under the influence of trust. Based on this, this paper proposes the following hypotheses:

*H7a*: Trust has a mediating effect between interactivity and purchase intention in tourism e-commerce live streaming.

*H7b*: Trust has a mediating effect between authenticity and purchase intention in tourism e-commerce live streaming.

*H7c*: Trust has a mediating effect between entertainment and purchase intention in tourism e-commerce live streaming.

## Methodology

### Questionnaire design and measurement

In order to ensure the reliability and validity of the questionnaire, this paper adopted the mature scale, and made appropriate modifications according to the characteristics of tourism e-commerce live streaming. All constructs were measured by Likert five-point scale, i.e., one means “strongly disagree” and five means “strongly agree,” and the larger the number, the higher the degree of agreement. The measurement of interactivity mainly referred to Liu et al. (2020a) and Wei et al. (2022). Items of authenticity referred to Tong (2017). The scale for entertainment was adapted from Chen and Lin (2018) and Lv et al. (2022).The measurement of flow experience mainly referred to Chen and Lin (2018). Items of trust referred to McKnight et al. (2002) and Chen et al. (2022c). The scale for purchase intention was adapted from Liu et al. (2013), Chen et al. (2017), and Liu et al. (2020b). The questionnaires were sent to experts in the field of tourism e-commerce live streaming for review. The initial questionnaire was formed after modification according to the experts’ suggestions. The initial questionnaires were sent to 50 respondents for pre-survey, and the final questionnaire was formed after modification based on the pre-survey results.

### Data collection and sample description

Questionnaires were distributed online and offline to avoid homologous deviation. The questionnaires were distributed online through the Wenjuanxing app, which is a professional online survey, evaluation and voting platform with nearly 50 million users in China (Liu et al., 2021f). The link of the questionnaire on Wenjuanxing app was shared through WeChat and QQ to expand the coverage of samples. Meanwhile, offline questionnaires were distributed to respondents by paper-based questionnaires. We selected individuals who had watched tourism e-commerce live streaming by the screening question (“Have you had the experience of watching tourism e-commerce live streaming in the past?”). Those people who had not watched tourism e-commerce live streaming were excluded. A total of 462 questionnaires were received, and 357 valid questionnaires were obtained by excluding invalid questionnaires with incomplete answers, illogical answers, and <1 min of online filling time, with an effective rate of 77.27%. Since the data for this study were obtained from both online and offline sources, there might be differences between the data obtained from the two sources. We tested the sample differences through a one-way ANOVA by summing the scores of all question items of each questionnaire. The ANOVA results show a value of *p* > 0.05, which indicates that there is no significant difference between the two groups of samples collected based on different routes. Therefore, the two groups of samples can be used as a whole sample.

The descriptive statistics of our survey samples are shown in Table 1. In terms of gender, there are more females than males, with 162 males (45.38%) and 195 females (54.62%). In terms of age, the group of 18–24 years old accounts for the largest proportion, and the next largest percentage is in the group of 25–30 years old. In terms of education level, there are more samples with bachelor degree or above. In terms of monthly income, those with monthly income of 5,000–10,000 yuan accounts for the largest proportion. In terms of online shopping experience, most of the samples have more than 3 years of online shopping experience. Overall, the samples in this study are representative of the tourism e-commerce live streaming consumers.

**Table 1:** Descriptive statistics of the study samples (*N* = 357).

Variable	Category	Frequency	Percentage (%)
Gender	Male	162	45.38
	Female	195	54.62
Age (years)	<18	23	6.44
	18–24	136	38.10
	25–30	129	36.13
	More than 30	69	19.33
Education level	High school and below	18	5.04
	Junior college	51	14.29
	Bachelor	159	44.54
	Master and above	129	36.13
Income (monthly/yuan)	Under 3,000	31	8.68
	3,000–5,000	92	25.77
	5,000-10,000	137	38.38
	10,000 or more	97	27.17
Online shopping experience (years)	<1	9	2.52
	1–3	52	14.57
	3–5	123	34.45
	More than 5	173	48.46

## Data analysis results

### Reliability analysis

Reliability reflects the stability and consistency of a scale. The greater is the reliability of a scale, the smaller is its standard error of measurement. In the Likert scale method, Cronbach’s alpha coefficient is the commonly used reliability test indicator. As can be seen from Table 2, the Cronbach’s alpha value for each construct in this study is above 0.7. This shows that the scale of this study has good reliability.

**Table 2:** Reliability analysis results.

Constructs	Items	Scales	Cronbach’s alpha
Interactivity (INT)	INT1	The tourism e-commerce live streaming allowed me to participate effectively	0.744
	INT2	I was able to communicate with the streamer timely while watching the tourism e-commerce live streaming	
	INT3	I was able to communicate with other viewers timely while watching the tourism e-commerce live streaming	
Authenticity (AUT)	AUT1	The information about the products or services presented in the tourism e-commerce live streaming was true	0.809
	AUT2	The tourism e-commerce live streaming presented the products or services from multiple perspectives	
	AUT3	The tourism e-commerce live streaming facilitated my in-depth understanding of the products or services.	
	AUT4	The direct experience of the products or services by the streamer deepened my understanding of the products or services	
Entertainment (ENT)	ENT1	The tourism e-commerce live streaming was interesting	0.825
	ENT2	The tourism e-commerce live streaming got me relaxed	
	ENT3	The tourism e-commerce live streaming gave me pleasure	
	ENT4	The tourism e-commerce live streaming was imaginative	
Flow experience (FE)	FE1	I was highly attentive (immersed) while watching this tourism e-commerce live streaming	0.880
	FE2	Sometimes I lose sight of what was happening around me while watching this tourism e-commerce live streaming	
	FE3	Sometimes I forgot what I was about to do while watching this tourism e-commerce live streaming	
	FE4	I felt in control when watching this tourism e-commerce live streaming	
	FE5	I felt happy when watching this tourism e-commerce live streaming	
Trust (TR)	TR1	I believed that the streamer was trustworthy	0.832
	TR2	I believed that the products or services information provided by the streamer were true	
	TR3	I believed that the products or services recommended by the streamer were of high quality	
	TR4	I trusted that the products or services I would receive would be the same as those shown on the tourism e-commerce live streaming	
Purchase intention (PI)	PI1	I intended to purchase products or services from this tourism e-commerce live streaming room	0.758
	PI2	I predicted that I would purchase products or services from this tourism e-commerce live streaming room	
	PI3	If there was a product or service that I would like to purchase, I would firstly purchase from this tourism e-commerce live streaming room	

### Validity analysis

Validity consists of convergent and discriminant validity. Convergent validity refers to a high degree of correlation between items, and discriminant validity refers to a low degree of correlation or the significant differences between constructs. Convergent validity is measured by the factor loading of each item, the composite reliability (CR) of the construct, and the average variance extracted (AVE) of the construct. It requires that factor loadings are preferably >0.5, combined reliability (CR) values are >0.6, and average variance extracted (AVE) values are >0.5 (Fornell and Larcker, 1981). According to Table 3, the factor loading of each item is >0.6, CR values are all above 0.7, and AVE values are >0.5. Therefore, the scale of this study has good convergent validity.

**Table 3:** Convergent validity analysis results.

Constructs	Items	Factor loadings	CR	AVE
Interactivity (INT)	INT1	0.768	0.754	0.506
	INT2	0.704		
	INT3	0.657		
Authenticity (AUT)	AUT1	0.717	0.809	0.515
	AUT2	0.691		
	AUT3	0.720		
	AUT4	0.742		
Entertainment (ENT)	ENT1	0.752	0.825	0.541
	ENT2	0.718		
	ENT3	0.768		
	ENT4	0.701		
Flow experience (FE)	FE1	0.752	0.880	0.595
	FE2	0.777		
	FE3	0.786		
	FE4	0.786		
	FE5	0.754		
Trust (TR)	TR1	0.741	0.832	0.554
	TR2	0.724		
	TR3	0.734		
	TR4	0.776		
Purchase intention (PI)	PI1	0.725	0.758	0.511
	PI2	0.725		
	PI3	0.693		

The discriminant validity of the scale is good if the square root of the AVE value of each construct is greater than the correlation coefficient between the constructs (Fornell and Larcker, 1981). The numbers on the diagonal in Table 4 are the square roots of the AVE values. It can be seen that the square root of each construct’s AVE value is greater than the correlation coefficient between its corresponding constructs. This shows that the discriminant validity of the scale in this study is good.

**Table 4:** Discriminant validity analysis results.

Constructs	M	SD	INT	AUT	ENT	FE	TR	PI
INT	3.725	0.555	**0.711**					
AUT	3.522	0.543	0.424^**^	**0.718**				
ENT	3.564	0.599	0.447^**^	0.252^**^	**0.736**			
FE	3.411	0.684	0.394^**^	0.345^**^	0.313^**^	**0.771**		
TR	3.667	0.599	0.253^**^	0.227^**^	0.235^**^	0.190^**^	**0.744**	
PI	3.652	0.584	0.453^**^	0.395^**^	0.347^**^	0.453^**^	0.375^**^^y^	**0.715**

** *p* < 0.01, the numbers in bold on the diagonal are the square roots of the AVE values.

### Common method bias and multicollinearity test

This study used a questionnaire method to collect data from the same subjects, so there was a possibility that the problem of common method bias may arise. In order to effectively control the generation of common method bias, Podsakoff et al. (2003) suggested the methods of ex ante procedural prevention and ex post statistical testing. In terms of ex ante prevention, the purpose of this study was stated in the first part of the questionnaire. We emphasized the anonymous completion of the questionnaire, avoided semantically ambiguous measurement questions, and selected consumers of tourism e-commerce live streaming in different provinces and cities. From the ex post statistical testing aspect, this study used the Harman one-way method to test the common method bias. The exploratory factor analysis was conducted by principal component analysis on all measured question items of the constructs of interactivity, authenticity, entertainment, flow experience, trust, and purchase intention without rotation. The results showed that the first principal component explained 29.773% of the total variance, which was less than the critical value of 50% (Podsakoff et al., 2003; Chen et al., 2022a). It can be seen that the common method bias problem in this study is not serious.

Multicollinearity refers to the inaccuracy of model estimation due to the presence of highly correlated relationships among the independent constructs in a linear regression model. Variance inflation factor (VIF) is one of the indicators to test for multicollinearity. In this study, the multicollinearity problem of the model was tested, and the results showed that none of the VIF values in this study was higher than 10. Therefore, there is no multicollinearity problem in this study.

### Hypothesis testing

We conducted structural equation modeling to verify the hypotheses. The indexes and evaluation criteria for evaluating the model fit (Wu, 2010) are shown in Table 5. The comparison shows that all the fit indicators meet the requirements, indicating that the model of this study has a good fit.

**Table 5:** Fitting of the study model.

Items	*χ*^2^/df	RMR	GFI	AGFI	NFI	RFI	TLI	CFI	RMSEA
Requirements	<3	<0.05	>0.9	>0.9	>0.9	>0.9	>0.9	>0.9	<0.08
Indicators	1.112	0.004	0.999	0.978	0.997	0.962	0.996	1.000	0.018

**Figure 2:**
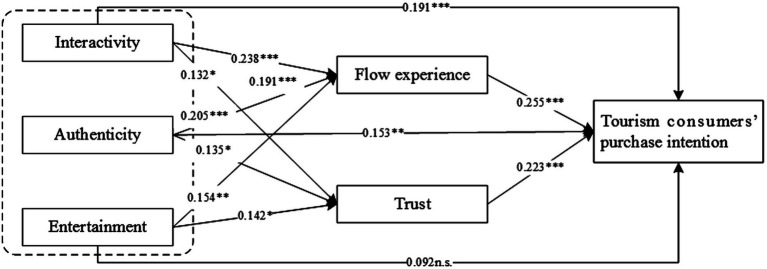
Path coefficient test results. ^*^p < 0.05, ^**^p < 0.01, and ^***^p < 0.001. n.s., not significant.

Table 6 demonstrates the path coefficients and hypotheses results in this study. Interactivity (*β* = 0.238, *p* < 0.001), authenticity (*β* = 0.205, *p* < 0.001), and entertainment (*β* = 0.154, *p* < 0.01) all positively influenced flow experience. Therefore, H1a, H2a, and H3a are supported. Interactivity (*β* = 0.132, *p* < 0.05), authenticity (*β* = 0.135, *p* < 0.05), and entertainment (*β* = 0.142, *p* < 0.05) all positively influenced trust. Therefore, H1b, H2b, and H3b are supported. Interactivity (*β* = 0.191, *p* < 0.001), authenticity (*β* = 0.153, *p* < 0.01), flow experience (*β* = 0.255, *p* < 0.001), and trust (*β* = 0.223, *p* < 0.001) all positively influenced purchase intention. Therefore, H1c, H2c, H4, and H5 are supported. The results of the data analysis show that entertainment does not influence purchase intention positively (*β* = 0.092, *p* > 0.05). Therefore, H3c is not supported. The results are shown in Figure 2.

**Table 6:** Structural equation model validation results.

Path	Standard path coefficient	Standard error	*T* value	*p* value	Hypothesis
INT → FE	0.238	0.069	4.233	^***^	H1a: supported
INT → TR	0.132	0.065	2.189	0.029	H1b: supported
INT → PI	0.191	0.054	3.676	^***^	H1c: supported
AUT → FE	0.205	0.066	3.933	^***^	H2a: supported
AUT → TR	0.135	0.061	2.417	0.016	H2b: supported
AUT → PI	0.153	0.051	3.198	0.001	H2c: supported
ENT → FE	0.154	0.060	2.929	0.003	H3a: supported
ENT → TR	0.142	0.056	2.507	0.012	H3b: supported
ENT → PI	0.092	0.047	1.921	0.055	H3c: not supported
FE → PI	0.255	0.04	5.4	^***^	H4: supported
TR → PI	0.223	0.043	5.052	^***^	H5: supported

*** *p* < 0.001.

In addition, a bootstrapping procedure with 5,000 samples was used to examine mediating effects (Shi et al., 2011; Zhou et al., 2015). The results are shown in Table 7. The effect of interactivity on purchase intention through the mediating effect of flow experience is 0.064 with 95% confidence interval excluding 0. The mediating effect is significant. Flow experience also mediates the both effects of authenticity and entertainment on purchase intention. The effect of interactivity on purchase intention through the mediating effect of trust is 0.031 with 95% confidence interval excluding 0. The mediating effect is significant. Trust also mediates the both effects of authenticity and entertainment on purchase intention. Therefore, H6a–H6c and H7a–H7c are supported.

**Table 7:** The mediation effects test analysis results.

Path	Estimated	*p*-value	Bias- corrected 95% confidence interval		Hypothesis
			Lower	Upper	
INT → FE → PI	0.064	0.000	0.035	0.105	H6a: supported
AUT → FE → PI	0.055	0.000	0.028	0.092	H6b: supported
ENT → FE → PI	0.041	0.008	0.012	0.076	H6c: supported
INT → TR → PI	0.031	0.028	0.003	0.068	H7a: supported
AUT → TR → PI	0.032	0.014	0.007	0.064	H7b: supported
ENT → TR → PI	0.033	0.009	0.008	0.070	H7c: supported

## Conclusion and implications

### Discussion and conclusion

This paper applied the S–O–R model to the study of consumers’ purchase intention in tourism e-commerce live streaming, focused on the influences of the tourism e-commerce live streaming features on consumers’ purchase intention, and analyzed the antecedent variables and paths of tourism consumers’ purchase intention through flow experience and trust. The following conclusions are drawn from the empirical research and analysis:

First, interactivity and authenticity of the tourism e-commerce live streaming features have positive effects on consumers’ purchase intention, but entertainment has insignificant effect on consumers’ purchase intention. Compared with the traditional online tourism marketing, tourism e-commerce live streaming is more immersive, as the streamer presents the tourism products to the consumers visually. The streamer introduces the tourism products, conducts live experience, and shares the experience. Tourism e-commerce live streaming creates a face-to-face shopping atmosphere, so that consumers can directly understand the advantages and disadvantages of tourism products. Tourism e-commerce streamers attract and retain consumers through real-time interaction and real product display, thus increasing the conversion rate (Li et al., 2021; Zhang et al., 2021a).The effect of entertainment on consumers’ purchase intention is not significant, which may be because when viewers watch tourism e-commerce live streaming for entertainment purposes, viewers will only stay in the viewing part and cannot directly generate purchase intention.

Second, the flow experience has a mediating role between the tourism e-commerce live streaming features and consumers’ purchase intention. The research results show that the interactivity, authenticity, and entertainment of tourism e-commerce live streaming positively affect flow experience, flow experience positively affects consumers’ purchase intention, and flow experience has a mediating role between the tourism e-commerce live streaming features and consumers’ purchase intention. The interactivity of tourism e-commerce live streaming allows consumers to communicate with the streamer and other consumers in both directions and then immerse themselves in the live streaming environment. The authenticity of tourism e-commerce live streaming can increase consumers’ interest in the products. The entertainment of tourism e-commerce live streaming can meet the pleasure psychology of consumers. The flow experience of tourism consumers makes them want to participate in the live streaming unconsciously and generate purchase intention under the stimulation and guidance of the streamer, which is consistent with the conclusions of Huang et al. (2021).

Finally, trust has a mediating role between the tourism e-commerce live streaming features and consumers’ purchase intention. The research results show that the interactivity, authenticity, and entertainment of tourism e-commerce live streaming positively affect trust, trust positively affects consumers’ purchase intention, and trust has a mediating role between the tourism e-commerce live streaming features and consumers’ purchase intention. There are risks of pictures being embellished in traditional online tourism marketing, so tourism consumers are often skeptical of tourism marketing. The interactivity of tourism e-commerce live streaming strengthens the emotional communication between consumers and streamers and brings the psychological distance closer. According to social exchange theory, consumers are more willing to trust the products recommended by streamers, thus increasing their purchase intentions (Cropanzano and Mitchell, 2005; Wei et al., 2022). In addition, the authenticity of tourism e-commerce live streaming weakens the risks of camera switching and excessive picture embellishment. Viewers can see the full information of live streaming scenes and products realistically, and every move of the streamer can be captured by viewers, increasing the credibility of online shopping. Therefore, the credibility of the information source positively affects consumers’ willingness to purchase (Tong, 2017). Finally, the entertainment of tourism e-commerce live streaming can increase consumers’ curiosity of the product and desire to participate, which leads to positive evaluation of the product and the streamer (Wongkitrungrueng and Assarut, 2020), thus increasing consumers’ purchase intentions.

### Research implications

Firstly, we have identified three unique features of tourism e-commerce live streaming features, namely interactivity, authenticity, and entertainment. Moreover, we studied the consumers’ purchase intention through the three features of the new media form. This provides a fresh perspective for the quantitative studies of tourism e-commerce live streaming. Previous studies have shown that e-commerce live streaming consumer’ purchase intentions can be influenced by live streaming strategy (Zhang et al., 2020), interaction (Li et al., 2021; Zhang et al., 2021b), and social presence (Ang et al., 2018; Chen and Liao, 2022). However, previous studies have mostly studied the impact of one feature of e-commerce live streaming on consumer’ purchase intention, without systematic and comprehensive studies, and they have not connected live streaming features with consumers’ perception (Sun et al., 2019; Deng et al., 2021; Qiu et al., 2021; Lin et al., 2022). This study confirmed that interactivity and authenticity of the tourism e-commerce live streaming features have positive effects on consumers’ purchase intention, but entertainment has insignificant effect on consumers’ purchase intention. It enriches the research content of tourism e-commerce live streaming.

Secondly, we offered theoretical insight into consumers’ purchase intention by employing the SOR model to tourism e-commerce live streaming research. A few studies have applied SOR model in the research of e-commerce live streaming (Xu et al., 2020; Guo J. et al., 2021), whereas, they have not paid attention to the tourism e-commerce live streaming. Since tourism products have special features (e.g., high unit price, low purchase frequency, intangibility, and non-transferability) that are different from general products (Xie et al., 2022), it is necessary to study on tourism e-commerce live streaming and the consumers’ psychology. The effectiveness of the SOR model in tourism e-commerce live streaming was confirmed, which provides a more profound and thorough understanding of the formation of tourism consumers’ purchase intention.

Finally, this study also examined the mediating effects of flow experience and trust on the relationship between tourism e-commerce live streaming features and consumers’ purchase intention, which contributes to research related to consumers’ purchase intention in live streaming commerce (Lu and Chen, 2021). To the best of our knowledge, in the existing literature, no research has examined the direct and mediating effect of flow experience in e-commerce live streaming. A few studies have provided empirical evidence for the positive effect of trust on e-commerce live streaming consumers’ purchase intention (Tong, 2017; Dong et al., 2022), whereas, they have not paid attention to the mediating effect of trust. The results of this study indicated that flow experience and trust partially mediates the impact of tourism e-commerce live streaming features on consumers’ purchase intention. It enriches the research content of emotional and cognitive reactions in tourism e-commerce live streaming.

### Practical implications

First of all, the positive roles of tourism e-commerce live streaming’s features on consumers’ perceptions point to the need for enterprises and streamers to invest resources in amplifying these three features when designing the live streaming. In terms of interactivity, the streamer should interact with consumers, enliven the atmosphere of the live streaming room, and make detailed and accurate answers to the questions raised by consumers. As one example, the streamer can design interactive lucky draws at different stages of the live-streaming process (Lv et al., 2022). In terms of authenticity, streamers should show the products in all aspects, strengthen the authenticity of the products and the consumers’ sense of live immersion, and create the feeling of offline shopping for consumers. Streamers can also effectively evaluate the products based on his or her own experience and provide consumers with purchase suggestions. In terms of entertainment, streamers can post some interesting content, discuss interesting entertainment topics, and hold a series of entertaining activities. For example, streamers can introduce tourism products in the form of sitcoms, or conduct role-play related to the tourism live streaming theme or destinations (Lv et al., 2022; Xie et al., 2022).

In addition, the flow experience has a mediating role between the tourism e-commerce live streaming features and consumers’ purchase intention. The research results show that flow experience positively affects consumers’ purchase intention, and flow experience has a mediating role between the tourism e-commerce live streaming features and consumers’ purchase intention. Therefore, the tourism e-commerce live streaming platform and streamers should enhance consumers’ flow experience in order to increase their purchase intention. First of all, the tourism e-commerce live streaming platform and streamers should seek to create attractive content that meets consumers’ expectations (Xu et al., 2019), and further compel them to continue watching and purchase. Additionally, emotional connections with the consumers through friendly words, passionate and immersive explanations and interactions are recommended strategies for streamers. Finally, when a consumer expresses confusion about the live streaming content, the streamer should give an accurate answer in time to satisfy the consumer’s curiosity about the tourism product. These methods enhance the consumer’s flow experience, thus increasing consumers’ purchase intentions.

Finally, trust has a mediating role between the tourism e-commerce live streaming features and consumers’ purchase intention. The research results show that trust positively affects consumers’ purchase intention, and trust has a mediating role between the tourism e-commerce live streaming features and consumers’ purchase intention. Therefore, the tourism e-commerce live streaming platform and streamers should enhance consumers’ trust in order to increase their purchase intention. First of all, a clear and comprehensive introduction of the products will help the consumers to enhance their perception of the consumption experience (Wongkitrungrueng et al., 2020), especially with tourism products. In addition, reliable streamers facilitate consumers’ trust (Ma et al., 2022). In order to assemble a group of high-quality streamers, organizations should establish strict recruitment standards. Finally, streamers should strictly control the quality of products according to their own expertise and eliminate unqualified products into the live streaming room, so as to enhance consumers’ trust and promote the formation of purchase intention.

### Limitations and further research

There are still some limitations in the study. Firstly, the features of tourism e-commerce live streaming are multifaceted, so future research can expand the features of tourism e-commerce live streaming by introducing factors such as the ease use of platform and platform usefulness to explore consumers’ psychology and behavior. Secondly, the features of tourism e-commerce live streaming affect consumers’ cognitive and emotional responses, but whether there are other mediating and moderating constructs in the influence mechanism need further investigation in the future. Finally, this study used a self-report questionnaire to collect data, and respondents might be influenced by various factors such as emotions and the environment. Therefore, the study results might be biased. In the future, multiple measurement methods can be tried to measure the constructs more accurately.

## Data availability statement

The raw data supporting the conclusions of this article will be made available by the authors, without undue reservation.

## Author contributions

XL contributed conception and design of the study, performed the statistical analysis, wrote the first draft, and revised the manuscript. LZ and QC organized the data collection. XL, LZ, and QC polished the manuscript. All authors contributed to the article and approved the submitted version.

## Funding

This work was supported by the Special Project of Zhejiang Provincial Social Science Foundation (21GXSZ063YBM), Soft Science Research Program of Zhejiang Province (2021C35045), Scientific Research Project of Zhejiang Education Department (Y202248666), Research Project on Higher Education of Zhejiang Gongshang University (Xgy22015), and the Key Project of Discipline Construction and Management of Zhejiang Gongshang University (2022).

## Conflict of interest

The authors declare that the research was conducted in the absence of any commercial or financial relationships that could be construed as a potential conflict of interest.

## Publisher’s note

All claims expressed in this article are solely those of the authors and do not necessarily represent those of their affiliated organizations, or those of the publisher, the editors and the reviewers. Any product that may be evaluated in this article, or claim that may be made by its manufacturer, is not guaranteed or endorsed by the publisher.
